# A new ML-based approach to enhance student engagement in online environment

**DOI:** 10.1371/journal.pone.0258788

**Published:** 2021-11-10

**Authors:** Sarra Ayouni, Fahima Hajjej, Mohamed Maddeh, Shaha Al-Otaibi

**Affiliations:** 1 Department of Information Systems, College of Computer and Information Sciences, Princess Nourah bint Abdulrahman University, Riyadh, Saudi Arabia; 2 College of Computer and Information Sciences, King Saud University, Riyadh, Saudi Arabia; Vellore Institute of Technology: VIT University, INDIA

## Abstract

The educational research is increasingly emphasizing the potential of student engagement and its impact on performance, retention and persistence. This construct has emerged as an important paradigm in the higher education field for many decades. However, evaluating and predicting the student’s engagement level in an online environment remains a challenge. The purpose of this study is to suggest an intelligent predictive system that predicts the student’s engagement level and then provides the students with feedback to enhance their motivation and dedication. Three categories of students are defined depending on their engagement level (Not Engaged, Passively Engaged, and Actively Engaged). We applied three different machine-learning algorithms, namely Decision Tree, Support Vector Machine and Artificial Neural Network, to students’ activities recorded in Learning Management System reports. The results demonstrate that machine learning algorithms could predict the student’s engagement level. In addition, according to the performance metrics of the different algorithms, the Artificial Neural Network has a greater accuracy rate (85%) compared to the Support Vector Machine (80%) and Decision Tree (75%) classification techniques. Based on these results, the intelligent predictive system sends feedback to the students and alerts the instructor once a student’s engagement level decreases. The instructor can identify the students’ difficulties during the course and motivate them through e-mail reminders, course messages, or scheduling an online meeting.

## Introduction

Over the past decade, internet-based learning is gaining more and more interest and popularity in higher education [1]. Many universities have implemented or planned to add large-scale online courses. Even traditional universities have adopted online learning to extend access and increase efficiency [2] by offering b-learning (hybrid classroom and online learning) courses. The prevalent acceptance of online learning has incited many researchers [3–7] to scrutinize the impact of online learning technology on the student’s engagement and student’s achievement. The construct of student engagement has been studied for many decades and still grasps the interest of many researchers in either traditional or online education settings [8, 9]. Many researchers link improving students’ achievement with the engaged time on tasks and activities [10]. However, student engagement has been considered more than academic engaged time and can be viewed as a multidimensional concept [8]. Although the majority of studies consider student engagement as one of the best indicators of learning [11], several researchers consider that there is no consensus on how to conceptualize and measure this concept. In this regard, Appleton *et al*. [12] explain that "literature" on engagement generally reflects a weak consensus on how to define, operationalize, and measure engagement.

Most studies of student engagement have focused on traditional, on-campus learning [6]. However, student engagement in an online environment becomes prevalent because of the drastic increase of this new mode of instruction [13] and thus the new challenges related to it. Some researchers interested in student engagement in online settings, tend to reduce this construct to the number of hours spent in online course [14] and others have used similar concepts and definitions as in traditional settings [15, 16]. Recently, some researchers addressed the problem of measuring and predicting student engagement based on data related to the student’s activities within a Learning Management System (LMS) or a Virtual Learning Environment (VLE) [9, 17, 18]. Learning management systems or Virtual learning environments (Moodle, Sakai, Blackboard, etc.) provide a huge amount of educational data related to students’ background, performance, behaviors and activities on the course. Valuable information and knowledge can be extracted from these educational data to support education-related stakeholders (i.e., instructors, students, decision-makers, etc.). Educational data are extensively used to predict the students’ performance, which is a well-established problem in Learning Analytics [19–21]. These data are stored in the e-learning systems (i.e., VLE or LMS), log activities and reports. Following that, the instructor can guide, motivate and engage the students through course messages, e-mails and meetings. Nevertheless, the important number of students in the section makes it arduous for the instructor to track all the students and predict the non-engaged students. Subsequently, an intelligent system that predicts student engagement by analyzing educational data stored in e-learning systems logs or reports is needed. Since the students’ engagement is a predictor of student academic performance [11, 21–23], it is of paramount importance to predict non-engaged students and try to make them more engaged and thus enhance their educational performance. Lee [24] consider that student engagement is a proximal outcome leading to distal outcomes such as better academic performance and reduced school dropout. In this context, we propose a predictive system that supports the instructor and the student as well. This system automatically identifies the level of student engagement in an online environment. Based on that prediction, the system automatically sends feedback to the student and an alert to the instructor at once. Then, the instructor can identify the students’ complications during the course and therefore motivate them through e-mail reminders, course messages, or scheduling an online meeting. When the students receive feedback regularly, they are more likely to work hard and increase their engagement. In order to predict the student engagement level, the authors propose to use student data activities within the LMS (e.g., Joining sessions, participating in forums and groups, course material access, etc.). All these data are recorded in several reports.

In the current study, the authors use data collected from different Blackboard reports to investigate student engagement in a course delivered online in Princess Nourah University (PNU). The Blackboard LMS stores course lectures, materials, and assessment information. It offers several kinds of reports, such as: (a) Course Activity Overview, (b) Session Activity Overview, (c) Student Overview for Single Course, (d) Overall, Summary of User Activity, (e) All User Activity in Content Areas, (f) User Activity in Forums, (g) User Activity in Groups, (h) Course Performance, (i) Course Coverage Report, and (j) Course User Participation. In the current study, we address the following research questions:

*Question 1*: Is it possible to classify a student as Actively Engaged (AE), Passively Engaged (PE) or Not Engaged (NE) based on students’ activities in the LMS?
*Question 2*: Which Machine Learning classification algorithm among SVM, ANN, and DT performs best in the proposed study?
*Question 3*: Does the feedback regularly sent to passively and not engaged students improve their engagement?

This study aims to predict student engagement level based on students’ activities recorded in the Blackboard reports. Different Machine Learning algorithms are tested to classify a student in one of the three categories: actively, passively, or not engaged according to its activities on an online course. These categories are determined using a new labelling method that considers both the student’s performance (i.e., GPA) and the teacher’s appreciation. The proposed system uses the most efficient predictive model in terms of accuracy, precision, recalling and F1-Score metrics to predict the student engagement level. It also helps to implement the best practices that can enhance engagement and improve students’ academic performance and achievement. It should be noted that this study differs from many others in that the focus is on the ability of our proposed system to classify students into three main categories (i.e., AE, PE and NE) according to the integrated data collected from different LMS reports. All the other studies focused on predicting a student is either totally engaged or not engaged. However, from personal experience, it is found that this is rarely the case and majority of the students are likely to be passively engaged. Our method permits a smooth transition from the two extreme states of engagement by predicting one more category (i.e., Passively Engaged). In addition, this study differs from others focusing on predicting student engagement based on student activities on LMS or VLE because it integrates data from different reports and considers many features and types of activities within the LMS. Some studies focused on a number of clicks [17] that could not reflect the real engagement level of the students. Others focused on data related to specific activities on an LMS (i.e., assignment submission activity [9]). Based on the prediction results, the proposed system sends feedback to the students and alerts the instructor once a student’s engagement level decreases. It is a priority for teachers to motivate the students to progress gradually, move up from one category to another, and encourage actively engaged students to keep the same pace of work.

The remainder of this article is structured as follows: Section 2 presents the related works. Section 3 introduces the motivation of the current work. Sections 4 and 5 describe our methodology and how Machine Learning algorithms are applied to student activities within an LMS and their evaluation. Section 6 presents to which extent the proposed intelligent predictive system permits preservation of student engagement level in online learning through a set of experiments carried out on three groups of students. Finally, Section 7 presents our conclusions.

### Related work

Online learning is becoming a more and more prevalent instruction mode in higher education that has been increasing steadily [1]. The growing acceptance and adoption of online learning has caused many researchers [15, 24, 25] to investigate the impact of technology on student engagement and learning outcomes. Most studies in the field have asserted the positive impact of information technology on student engagement [15, 26]. Some studies found that asynchronous learning allows learners to develop higher-order skills such as problem-solving, analysis, synthesis, judgment, collaboration and stimulation. Duderstadt et al. [15] state that online learning can stimulate students to use higher-order skills when it is implemented through active, inquiry-based learning pedagogies, online learning can stimulate students to use higher-order skills.

Furthermore, in online learning, students tend to work more collaboratively, which is an important component of student engagement [27]. In [6], the authors explained that adopting online learning has enhanced accessibility to higher education and therefore created the demand for greater accountability and evidence of effectiveness in learning, and one of the primary predictors of effective online learning is student engagement. Therefore, it is crucial to focus on student engagement in online environment to ensure the effectiveness of such educational style [28–30]. Dabbagh and Kitsantas [30] confirmed that online learning allows learners to communicate with other learners and instructors, use technology easily and especially have a sense of belonging to the learning environment. Golladay et al. [16] defines engagement in an online environment as the time spent by faculty to prepare lessons and communicate with motivated learners and the time spent by learners to take online classes using the advantages of technology. Dixson [23] suggest that engagement indicators in online learning consist of skills, participation, performance and emotion. Skills indicators are presented by the importance of learning style as the regular attendance, listening carefully, and taking summaries and notes. Participation consists of the interaction between different peers using discussion, sending messages and chatting. Performance is studied by grades assigned to exams, quizzes and tests. These researches consider several important students’ characteristics that can be accounted for when evaluating their engagement at the course and activity levels, such as communication skills, cooperative work and motivation. However, the majority of these studies [1, 26, 29] used survey/instrument, observation/interview, and self-reporting to evaluate student engagement in online learning at the end of the course or activity. While this information is often useful, it also presents significant limitations. Students reporting their academic experience after completing a course or an activity might be inaccurate.

An online learning environment provides a vast amount of data that can be collected. Each student action (reading files, participating in forums, sending messages, or visiting recommended links, for example) leaves a digital fingerprint that can be analyzed using machine learning and learning analytics (LA). In reviewing LA literature, many existing studies focused on predicting students’ outcomes [31–33], students at risk [34–36], students’ performance [37–41]. Some other studies used deep learning paradigm to measure teaching effectiveness by establishing sentiment classification scheme using student evaluation of teaching [42].

Recently, some studies applied LA to predict student engagement in online learning environment based on collected data from LMS or VLE. Mushtaq et al. [17] used three different variants of decision tree algorithms to predict student engagement on three input variables, including the highest education Level, final results, score on the assessment, and the number of clicks on Virtual Learning environment activities. Only low-engaged students were detected. They developed a dashboard for the instructor to provide additional interventions for students in advance of their final exam. Motz et al. [9] examined some student activities on LMS, especially those related to assignments. They used the K-means clustering approach to classify the courses. Then they applied mixed-effects logistic models on the different clusters they obtained in order to investigate the relationship between features of student activity and instructors’ rating of student engagement. They found that these activities could provide valid and useful indicators of student engagement compared to instructor engagement rating. Nesrin and Manal in [43] used Decision Trees, Random Forest and Grading Boosting Machine to predict student performance based on student and parent engagement. They considered data on LMS and several other external data related to demographic, academic backgrounds and parent involvement. They found that parent engagement features increase student performance.

Several studies have applied learning analytics to predict student performance using different machine learning algorithms. Most researchers agree on the impact of student engagement on their performance. However, few research studies have explored LA to predict student engagement in relation to their performance and did not explore how to enhance and motivate students and keep them engaged in the online course.

### Motivation for the study

Traditionally, the teacher would evaluate students’ level of engagement based on their participation and performance in class. Due to the lockdown caused by the Covid-19 pandemic, many universities have been shifted to online teaching-learning. Accessing learning resources, submitting assignments and exams, communicating with the instructor as well as with peers are all fulfilled online during the pandemic period. Hence, one might be led to assume that all these activities on LMS can provide indicators to measure student engagement level. Student engagement has been identified as a complex and multidimensional construct [44, 45] that can include behavioral, cognitive, social and emotional dimensions. The last dimension is relevant to the student engagement construct, but it is elusive, especially in an online environment where it is difficult to capture and measure the emotional connection between students and teachers. The main purpose of the current study is to use LMS data to identify student engagement levels in terms of behavioral, cognitive and social dimensions.

In the Kingdom of Saudi Arabia (KSA), 28 public universities use LMSs as a part of their educational process. PNU is one of the 89% of public universities, and it uses the Blackboard LMS [46]. During the pandemic, all courses are shifted online (i.e., from March 2020) [18], and the teaching process is conducted entirely online except for some labs that remain on-campus. It is difficult for the instructor to identify and track non-engaged students in online settings based on personnel experience. Hence, a system that automatically detects student engagement levels is needed to support both the instructor and the student. Such a system could allow the instructors to make an adjustment to their pedagogical approach or teaching materials when it is apparent that the students are not engaging fully. The students could also improve their engagement level when they regularly receive feedback and encouragement. To the best of our knowledge, in KSA, there is no study interested in evaluating student engagement. Given the importance of student engagement construct, few researchers have attempted to derive valid and reliable measurements related to this context.

## Methodology

The most widely used method to measure student engagement is the use of surveys and questionnaires, thus through information reported by the students themselves. Other methods include checklists and rating scales completed by teachers, observations, and case studies [26]. It is essential to define the scope of student engagement in a specific context in order to be able to assess it effectively [9, 17, 43, 47]. In this article, the dynamic nature of the behavioral, social, and cognitive dimensions of student engagement is captured based on student activities, and actions on a course from LMS reports [48]. To answer the first research question (Question1: Is it possible to classify the student as Actively Engaged (AE), Passively Engaged (PE) or Not Engaged (NE) based on students’ activities on a course that are recorded in the LMS reports?), we developed a method that classifies a student engagement into three levels. This method is based on the student Grade Point Average (GPA) and instructor’s appreciation. We added the categorical target variable (class) representing the student engagement level based on their GPA. It has three categories AE, PE and NE that correspond to [A+, B+], [B, C], and [D+, F] grade ranges, respectively. After that, the resulted student engagement levels were adjusted and validated based on the instructor’s recommendation and appreciation. The result of this step constitutes the labelling phase of the data set preparation. To answer the second question (Question2: Which Machine Learning classification algorithm among SVM, ANN and DT performs best our study?), we developed three prediction classification models, namely Decision Tree Classification (DT), Support Vector Machine (SVM) and Artificial Neural Network (ANN). In the literature, different Machine Learning algorithms were used in different domains depending on the target application [49, 50]. In this study, we selected the three Machine Learning models (i.e., DT, SVM and ANN) for their suitability to our target application. Decision Tree Classification as a Machine learning technique is widely used for its simplicity and its easy interpretation. In our study, we used DT to determine the most important features in a student engagement prediction model. SVM is a classification technique suitable for small datasets, especially those with a small number of features. The ANN technique is used for its ability to handle small datasets. We calculated and compared the performance of these three selected models using the recall, precision, accuracy, and F1-Score metrics. To answer the third question (Question3: Does feedback send regularly to passively and not engage students improve their engagement?), we performed a set of experiments on three different groups of students. One group was provided by regular feedback after the completion of each learning module according to their engagement level prediction. Students of another group were provided by feedback on some specific weeks. A third group was not provided with feedback. After that, those groups were compared to each other in terms of engagement level (i.e., provided by the prediction model).

### Models building and evaluation

As mentioned previously, we aim to predict student engagement level to be either not engaged, passively engaged or actively engaged. As engagement directly affects the students’ results [51–53], the goal of the proposed prediction system is to evaluate their engagement and motivate them to progress continuously and move up from one category to another; it also helps engaged students to keep the same pace of work.

#### Data collection and description

In this study, we used the data of 360 students enrolled in College of Computer and Information Sciences at PNU. We obtained the ethical approval of the current study from the Institution Review Board of PNU (IRB Log Number: 21–0256). To get the most value out of the study and for accurate results, the authors excluded data rows related to dropouts and course withdrawals. As a result, we came with a dataset of 348 data rows and 10 columns (i.e., the first column presents the student ID, the 9 others are student engagement indicators). The data related to students was collected for three months: February, March and April 2020. We exported several reports derived from virtual class sessions, course messages, discussion boards and the different reports selected in the evaluation content area as Student single course activity, User participation report, User Activity in Forums, etc. We used the following derived indicators from these reports as features in our dataset: the *Total Logins* indicator represents the number of times a student entered their username and password to login to a particular section course in the Blackboard. The *Join session* indicator represents the number of times a student accesses a particular synchronous virtual class. The *Time spent on session attendance* indicator determines the total hours that a student spends in virtual class during the period of examination. The *Number of items* indicator represents the different items that a student selects as assignment submission, course materials download, quizzes submission, etc. The *Time spent on all items* indicator is the total period that a student is logged in to the Blackboard and inside the specific section (not just to access the Blackboard for a particular class). The *Number of Activities inside the content area* is captured each time a student selects an item in the Blackboard (i.e., learning module, assignment, quiz, etc.) The *Communication percentage* includes discussion between student/instructor and student/student (i.e., sent messages between tutor and students) and participation in forum (i.e., thread, post, reply and read). The *Number of forum clicks* represents the number of times students click in the area of the forum in which they did not participate but only read posted discussions. In the studied course, students are divided into groups. The *Total number of students’ activities in-group* represents the participation ratio of students within the group. The collected indicators from the Blackboard reports could not be directly used as inputs in the Machine Learning models. Thus, the input features of the current study needed to be integrated into a single file in an acceptable format for machine learning algorithms.

#### Dataset labelling

Before developing the predictive models, we established a label of engagement level and a student can be classified into three main categories (NE), (PE) and (AE). In the literature, there is no clear consensus on how to find the categories of student engagement [9]. That is why we need to develop our own labelling method. Substantial body of the literature considers learner engagement is a good indicator of positive learning outcomes [25, 54]. Based on this fact, we propose to classify student engagement according to their GPA, thereafter, teachers will validate the obtained result. So, student engagement is identified based on students’ GPA and the feedback of their teachers. We divided the set of examples into three groups: AE, PE and NE having respectively grades between [A+, B+], [B, C] and [D+, F]. The results of the first step are shown to teachers to be checked. Based on teachers’ appreciation and recommendation some students were moved from one category to another. For example, some students with high GPA and classified as actively engaged were moved to passively engaged. In addition, some students with C+ grade were judged to be actively engaged by their teachers. Four teachers were involved in validating and adjusting the engagement levels of their corresponding students. The different statistics related to the cleansed dataset are shown in Table 1.

**Table 1:** Statistics of the set of examples.

Category	Number of Students	Number of adjusted Students	Adjustment Rate
AE	112	98	12%
PE	191	199	4%
NE	45	51	13%

#### Building and testing the predictive models

After collecting, integrating and preparing student data activities within the Blackboard LMS, we trained three predictive models (DT, SVM and ANN). The input features for the training are the nine engagement indicators extracted from the blackboard reports (these indicators are illustrated in section 5.1), and the target variable is the predicted student engagement level (AE, PE and NE). Each model is developed using 10-fold cross-validation, where nine subsets are used for training, and the remaining subset is used for testing.

#### Models evaluation

The performance of the machine learning models is measured using the following metrics: the precision represents classifier predictive power; the accuracy represents classifier effectiveness; the recall represents classifier sensitivity and the F1-Score that conveys the balance between the recall and the precision. These measures are given in Eqs (1), (2), (3) and (4), respectively.


Precision=TP/((FP+TP)) (1)

Accuracy=(TP+TN)/((FP+FN+TP+TN)) (2)

Recall=TP/((FN+TP)) (3)

F1-Score=2×Recall×Precision/(Recall+Precision) (4)

Such that: TP, TN, FP, and FN refer to True positive, True Negative, False Positive, and False Negative, respectively. The performance of the three machine learning models is shown in Table 2.

**Table 2:** Models’ performance.

	Accuracy	Recall	Precision	F1-score
**ANN**	0.85	0.89	0.81	0.84
**DT**	0.80	0.81	0.82	0.81
**SVM**	0.75	0.71	0.81	0.73

After evaluating the three predictive models, we aim to determine which one is the most suitable for predicting student engagement level and to identify which student activities are more significant. The results reported in Table 2 show that it is possible to predict student engagement level using machine learning models.

**Fig 1:**
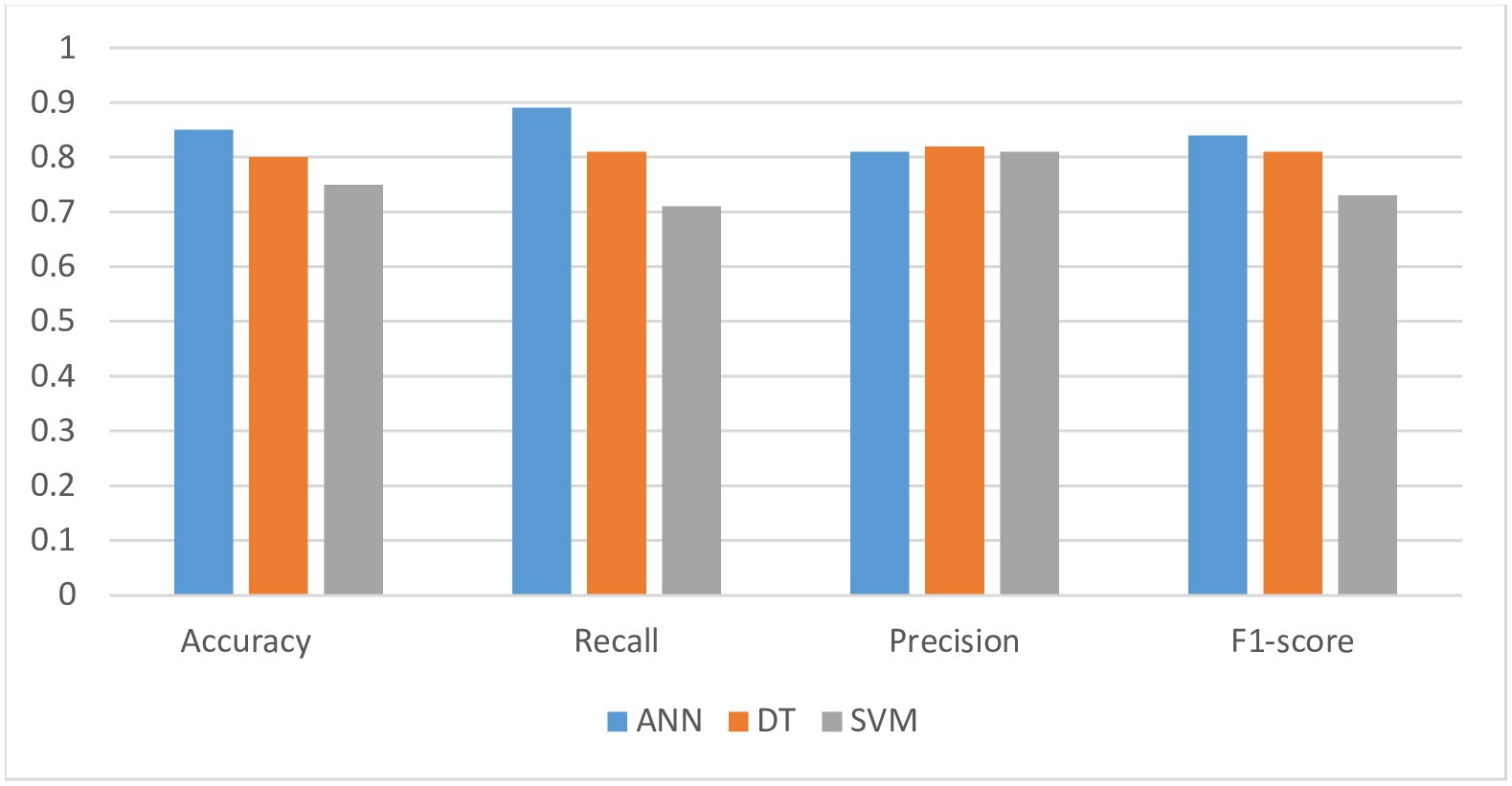
Performance metrics of ANN, DT and SVM.

Fig 1 shows the comparison of the three models performance. The Precision, Recall, F1-Score, and Accuracy metrics demonstrate an acceptable performance of all the applied classifiers. Therefore, we confirm the usability of these models (ANN, DT and SVM) to predict the students’ engagement level within an online learning environment. As shown in Fig 1, it can be seen that the ANN model outperforms the other models in term of Accuracy, Recall and F1-score. These findings are mainly supported by many researchers predicting student performance (e.g., [19, 20, 41]). In addition, it should be noted that DT classification model outperforms the SVM and ANN model in terms of Precision.

**Fig 2:**
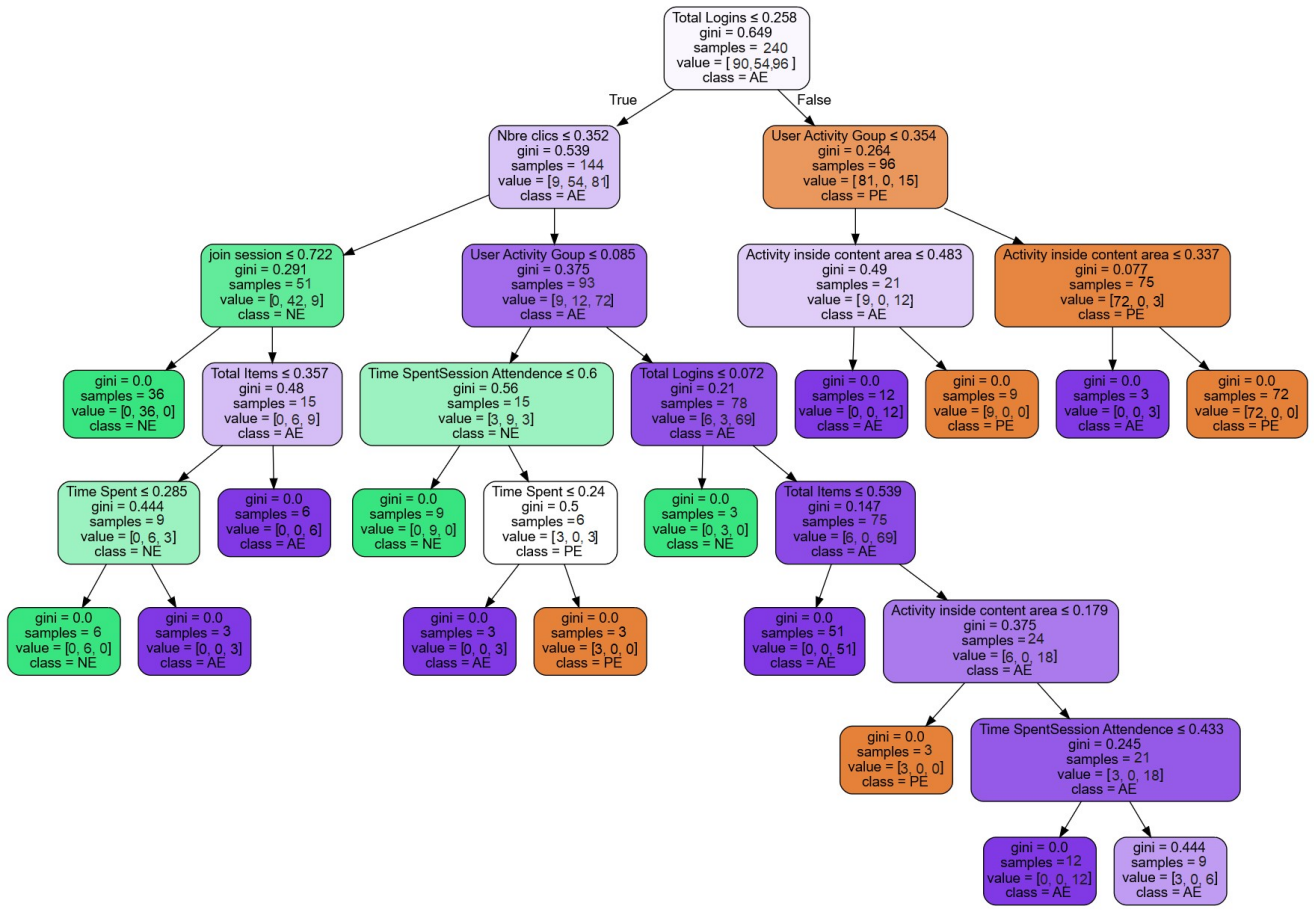
The decision tree generated by the DT classifier.

To find which online learning activities are more significant for student engagement prediction, we propose to analyze the results of the decision tree. To do so, the Features-Importance (i.e., calculated as the decrease in node impurity weighted by the probability of reaching that node.) is applied to the DT classifier in order to determine the most important features that affect the level of engagement. The decision tree obtained from the used dataset, shown in Fig 2, is easy to read and interpret. Several interesting observations can be drawn from the DT classifier: the *Total login*, *Number of clicks*, *Join sessions*, *User activities group*, *Activities Inside content area* features are the most important predictors of student engagement. This can be explained by the high Gini and Importance values they have (as shown in Table 3). Other features, such as time spent, total items and time spent Session Attendance are not that important predictors. In addition, the results show that actively engaged students (AE) are likely to have high number of clicks and participate more in activities inside the course as well as inside the group. Therefore, these AE students tend to interact more with other students. In contrast, passively engaged students (PE) have fewer clicks inside course area and participate less in group activities; however, they are joining course sessions and spend more time than not engaged students (NE).

**Table 3:** Features’ importance.

Feature	Importance
Total Logins	0.310349
Activity inside content area	0.294501
Nbr. Clicks	0.137109
join session	0.100937
User Activity Group	0.074397
Total Items	0.034525
Time Spent	0.031743
Time Spent Session Attendance	0.016438

### Evaluation of the proposed predictive system

Based on the selected model that best fits our study (i.e., ANN), our proposed system will send feedback to students with low engagement or those whose engagement has become increasingly low. The main purpose of sending such feedback is to evaluate the effectiveness of the proposed system. To do so, we put forward the following hypotheses that we intend to approve or disapprove:

**H1**: The student whose engagement level is evaluated and received feedback will participate and will be more engaged than the students who did not receive feedback.
**H2**: The more feedback a student gets, the more engaged he/she is.

We carried out series of experiments on three different groups of students. The system provided only two groups and received feedback at different momentum. The experiment concerns a sample of students in level 4 during the first semester 2020–2021. A total of 121 students were divided into three groups. Each group represents a specific target for the experiment as follows:

- *The first group (G1)*: consists of 40 students. This group is using an LMS without the predictive system.
- *The second group (G2)*: consists of 41 students. This group uses an LMS and is provided by the predictive system to keep track of the students’ engagement level.
- *The third group (G3)*: Consists of 40 students. This group uses an LMS and is provided by the predictive system.

**Fig 3:**
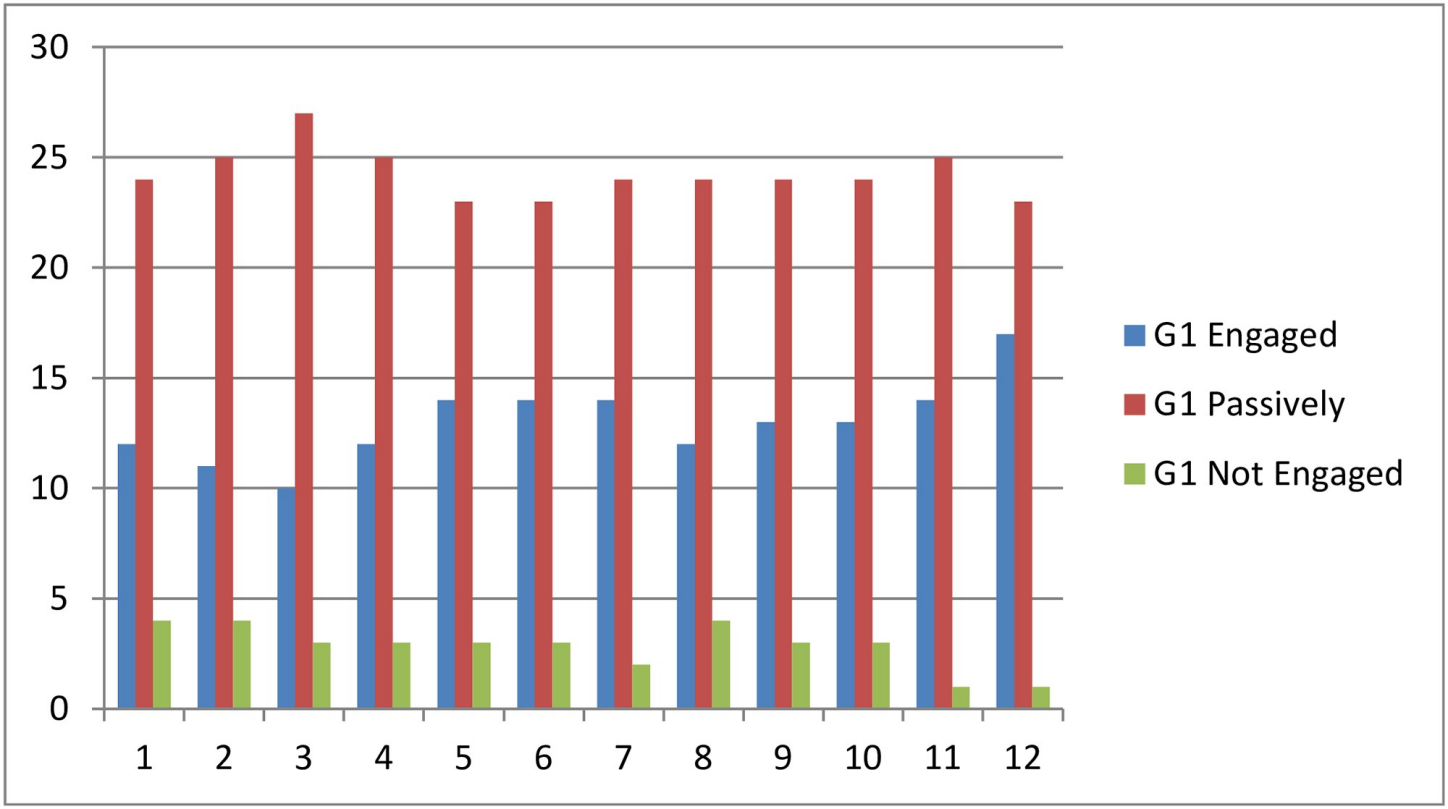
G1 engagement level.

The three groups study the same course and are taught by the same teacher. Using the predictive system, students from the second group G2 will receive immediate feedback after the completion of each learning module, and students from the third group G3 will receive feedback on specific dates (week 4, week 8 and week 14) which correspond, respectively, to the 4th, 9th and 12th learning modules. The experiments were performed during one semester (i.e., 15 weeks). Fig 3 shows the evolution of the number of students in group G1 according to their engagement level over the semester. It can be seen that most of the students are passively engaged, but on week 14 (i.e., learning module 12), the number of engaged students is increasing, and the number of not engaged students is decreasing.

**Fig 4:**
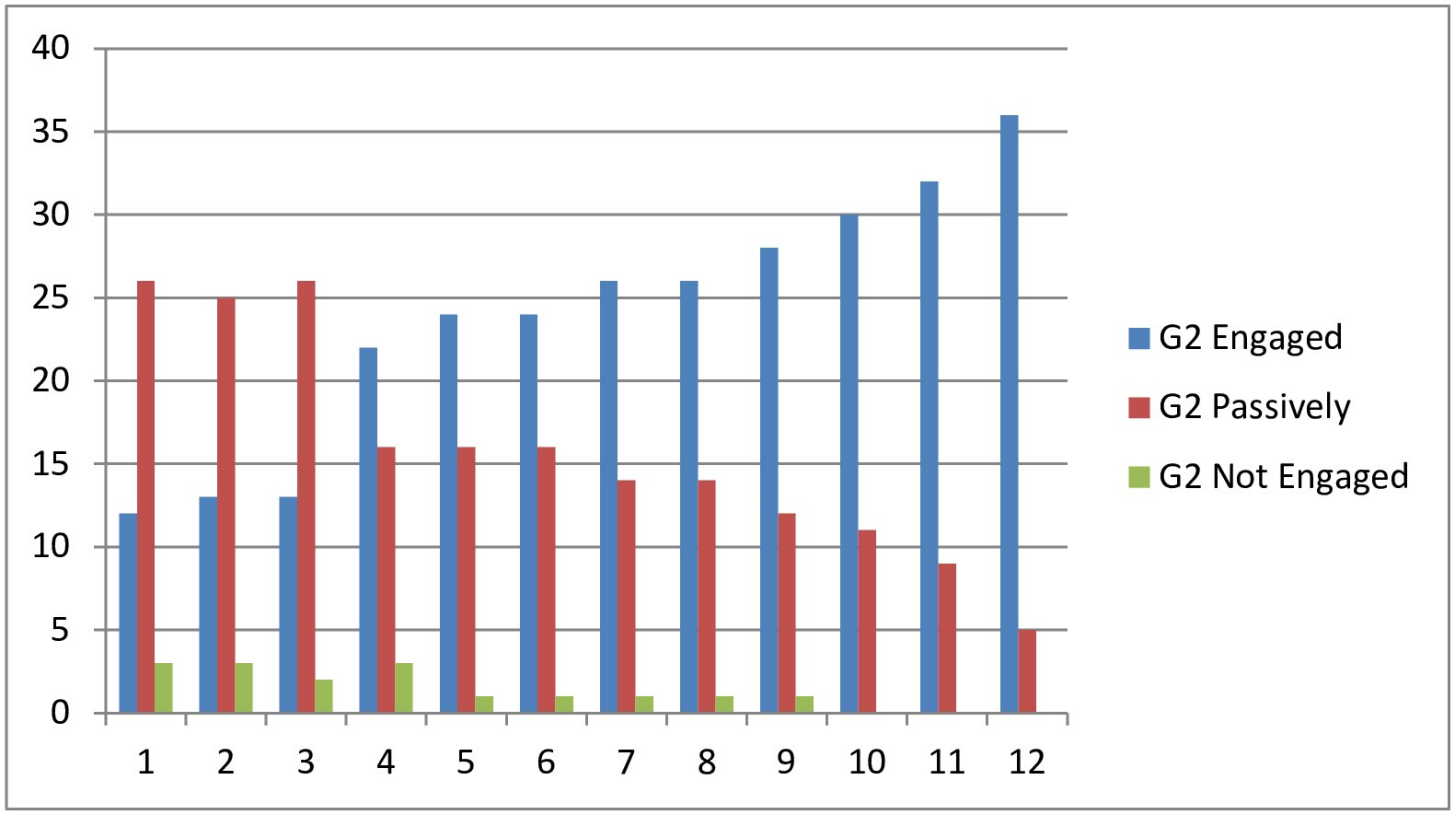
G2 engagement level.

Fig 4 shows the evolution of student’s number in group G2 according to their engagement level over the semester. It can be observed that the number of engaged students is increasing over the 12 learning modules of the course. Many students who were passively engaged and not engaged are becoming more and more engaged as the feedback number is increasing.

**Fig 5:**
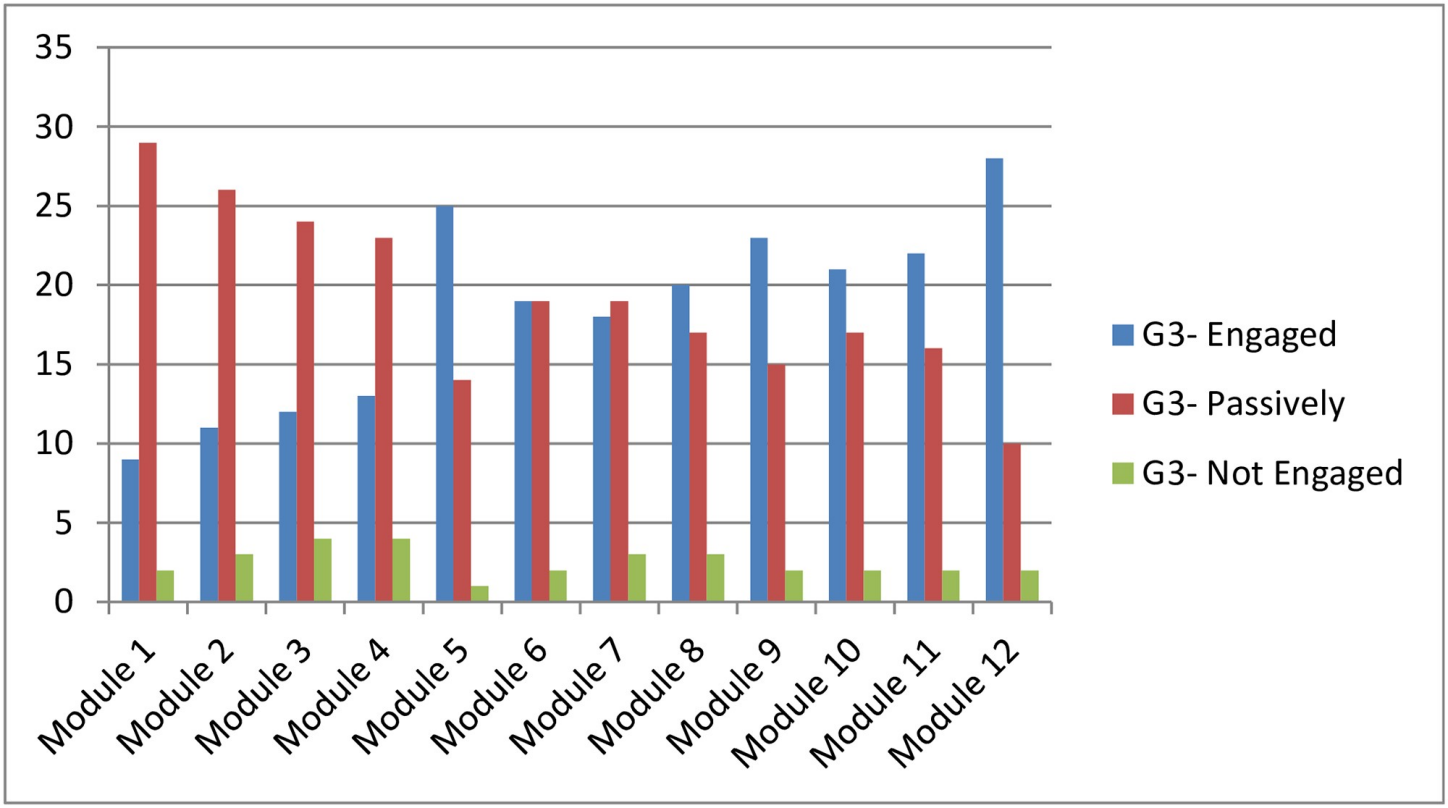
G3 engagement level.

Fig 5 shows the evolution of the number of students in group G3 according to their engagement level over the semester. The number of engaged students is increasing in learning modules 5, 9 and 12. This group of students were provided with feedback on week 4, 9 and 14 that correspond respectively to the learning modules 4, 8 and 11. It can be observed that the students are becoming more and more engaged after receiving feedback.

In the first group G1, the number of actively engaged students is relatively increased only before the completion of the course shortly before the final exam. In contrast, the two other groups that received feedback improved their engagement level over the semester. The module 12 is taught on week 14 where 80.48% of the students in G2 are becoming Engaged, 17.07% of the students are passively engaged and only 2.43% are not engaged. On week 14, only 62.5% of the students in G3 are engaged, 29.27% are passively engaged and 7.5% are not engaged. Based on these results, we can deduce that the more feedback a student receive the more engaged is and H2 is supported.

**Fig 6:**
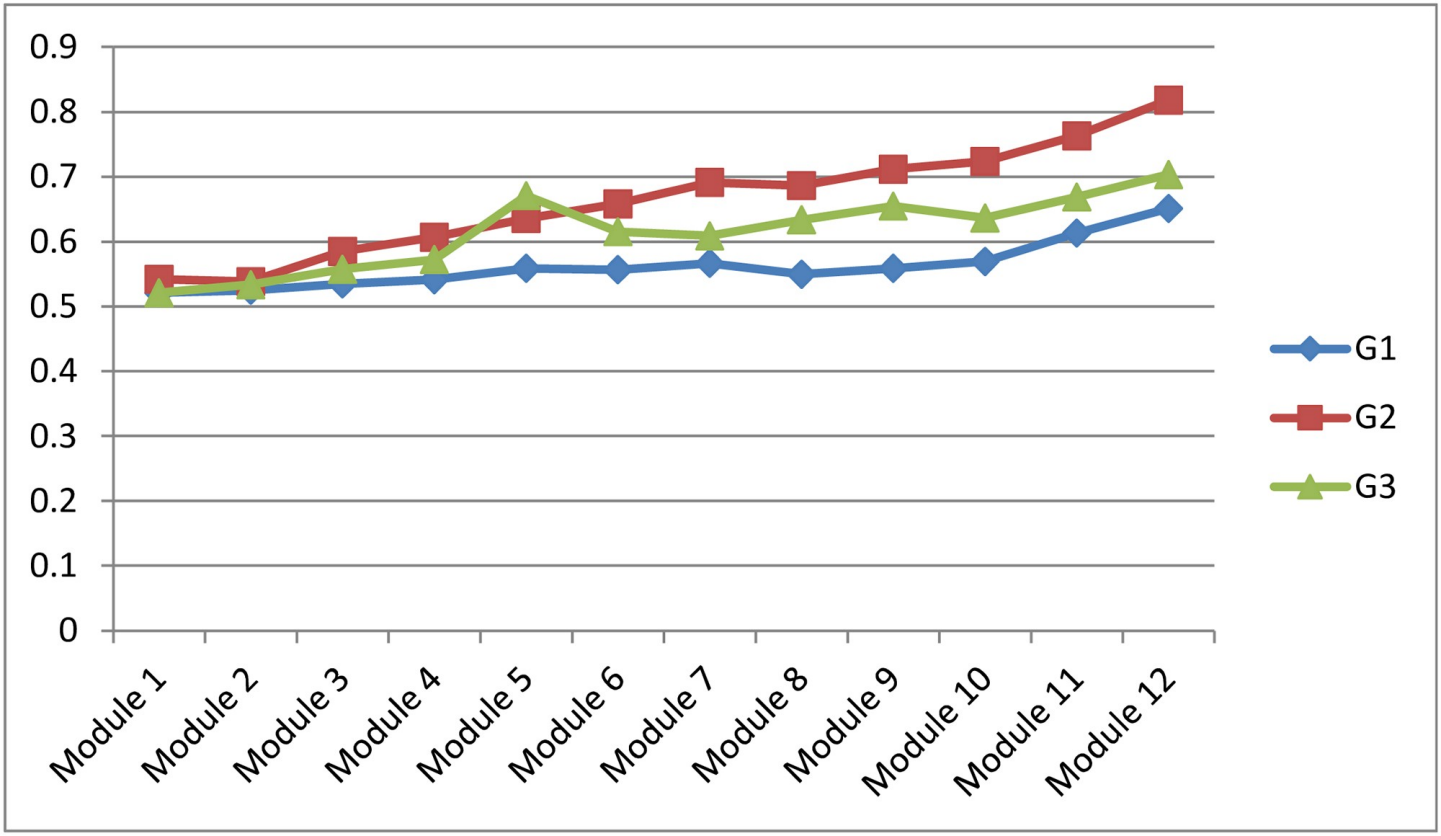
Student engagement evolution of G1, G2 and G3.

Fig 6 shows the variation of the mean values of students’ engagement level of the three groups over the 12 learning modules delivered along the semester. It can be seen that the overall engagement level of all the students is increasing over time and reaches its maximum by week 14. However, there is a notable difference between students’ engagement level of the three groups. The second group of students who received feedback after the completion of each learning module are more engaged than the others. The students in the third group are more engaged than students of the first group who did not use the predictive system. This result confirms the first hypothesis H1 asserting that “student whose engagement level is measured and has received feedback will participate and will be more engaged than students who did not receive feedback”.

Hence, the more the students’ progress in the online learning process and receive more feedback, the more significant improvement in their engagement level and therefore their performance. It can, therefore, be claimed that the proposed intelligent predictive system encourages the students to be more involved and dedicated.

## Conclusion and discussion

This study aims to focus on two main targets: the first is to evaluate student engagement level by building a well-performed prediction algorithm and the second is to increase students’ engagement by motivating them and keeping them aware of their dedication through feedback. The authors collected data from LMS reports and formatted them in a suitable format usable by ML algorithms. Further, they applied three different ML classifiers and evaluated them using a set of performance metrics. The results confirm the effectiveness of classification modeling in predicting student engagement. In addition, the study shows that ANN outperforms the other models with an accuracy rate of 85%. Nevertheless, based on the DT algorithm applied to the collected dataset, we identified the most important features as a predictor of student engagement level.

The proposed system may help keep students engaged significantly with the disruption in education provision caused by the COVID-19 pandemic; maintaining students engaged during the lockdown seems challenging and requires careful attention to contend with low achievement, alienation, and high dropout rates. The results of a series of experiments conducted on three different groups of students taking the same course taught by the same instructor confirm our hypotheses regarding the positive effect of the proposed intelligent predictive system on student engagement. This study shows that measuring students’ level of engagement and providing them with feedback at the appropriate moment ensure students focus on the course, which will in turn support student performance and experience.

In the current study, the prediction of student engagement level is based on students’ activities inside the same online course. Because of the complexity of student engagement construct, many other indicators such as course design, teaching style and other factors external to the course should be investigated. Future work should consider these factors in the prediction of student engagement as well as student outcomes. We will also study the impact of teaching quality and pedagogical practice to enhance engagement and improve the teaching process.

## Supporting information

S1 Dataset (CSV)
